# American Rhinological Association in Europe

**Published:** 1889-10

**Authors:** R. S. Knode

**Affiliations:** Secretary; Omaha, Neb.


Omaha, Neb., July 26, 1889.

Dear Doctor:—Owing to the absence of a number of the fellows of the American Rhinological Association in Europe, and to the Pacific Coast, the Annual Meeting will be postponed until October 9th, 10th and 11th, 1889, at which time it will be held at the Palmer House, Chicago, Ill.

By order of the President.

R. S. KNODE, Secretary.

